# An analysis of conventional and modern packaging approaches for cut flowers: a review article

**DOI:** 10.3389/fpls.2024.1371100

**Published:** 2024-03-27

**Authors:** Nahed M. Rashed, Saba Ambreen Memon, Saleh M. Al Turki, Tarek A. Shalaby, Mohamed M. El-Mogy

**Affiliations:** ^1^ Department of Arid Land Agriculture, College of Agricultural and Food Science, King Faisal University, Al-Ahsa, Saudi Arabia; ^2^ Horticulture Department, Faculty of Agriculture. Damietta University, Damietta, Egypt; ^3^ Horticulture Department, Faculty of Crop Production, Sindh Agriculture University, Tandojam, Pakistan; ^4^ Horticulture Department, Faculty of Agriculture, Kafrelsheikh University, Kafr El-Sheikh, Egypt; ^5^ Vegetable Crops Department, Faculty of Agriculture, Cairo University, Giza, Egypt

**Keywords:** modified atmosphere packaging, controlled atmosphere packaging, nanotechnology, intelligent packaging, postharvest, shelf-life, storage

## Abstract

Fresh-cut flowers are considered to be one of the most delicate and challenging commercial crops. It is important to take into consideration how to minimize loss during storage and transportation when preserving cut flowers. Many impinging (bad effect) forces can interact to shorten the flowers’ vase life. In the flower industry, effective methods need to be developed to extend freshly cut flowers’ life. Fresh-cut flowers’ vase life can be shortened by a variety of interlocking causes. The flower industry must develop new techniques to extend the flowers’ vase lifespan. This review provides comprehensive, up-to-date information on classical, modified atmosphere packaging (MAP), and controlled atmosphere packaging (CAP) displays. According to this review, a promising packaging technique for fresh flowers can be achieved through smart packaging. A smart package is one that incorporates new technology to increase its functionality. This combines active packaging, nanotechnology, and intelligence. This technology makes it easier to keep an eye on the environmental variables that exist around the packaged flowers to enhance their quality. This article offers a comprehensive overview of creative flower-saving packaging ideas that reduce flower losses and assist growers in handling more effectively their flower inventory. To guarantee the quality of flowers throughout the marketing chain, innovative packaging techniques and advanced packaging technologies should be adopted to understand various package performances. This will provide the consumer with cut flowers of standard quality. Furthermore, sustainable packaging is achieved with circular packaging. We can significantly reduce packaging waste’s environmental impact by designing reused or recyclable packaging.

## 1 Introduction

Almost all cut flowers have short vase life and fast quality deterioration because of their ephemeral nature. Postharvest practices are crucial in maintaining both the quality and longevity of cut flowers, owing to their ephemeral nature (Hassan and Schmidt, 2004). After harvest, cut flowers have several factors that affect their quality. These factors can be pre- or postharvest, underscoring the importance of effective flower management in extending their lifespan (Ali et al., 2022). A variety of factors contribute to harvesting, grading, precooling, conditioning, pulsation, bunching, wrapping, packaging, storing, and transporting (Belay et al., 2016; Hassan et al., 2020a; Kumar et al., 2022). Proper management of all these stages can significantly enhance the vase life and quality of flowers (Hassan et al., 2014; Hassan et al., 2020b). In addition to reducing the marketability and quality of harvested flowers, they also limit the commercial value of the flowers (Mazrou et al., 2022).

Furthermore, the preservation of cut flower quality postharvest has consistently been a subject of significant scientific interest.

Due to increased respiration and transpiration during flower handling, transportation, and export, the flowers undergo physiological stress. This expedites the deterioration of their quality (Belay et al., 2016). Furthermore, packaging serves to preserve flower quality during transport, export, and storage, thereby increasing their chances of safety. A direct consequence of this is an improved marketing and distribution network. Losses in the cut flower industry are estimated to be approximately 20%–40% due to inefficient postharvest packaging and storage; hence, investing in this area could potentially yield higher returns than expanding production. Also, cut flowers after harvest will wilt; will be abscised, discolored, and dehydrated; and will brown and succumb to senescence. An increased CO_2_ and reduced O_2_ atmosphere during storage can mitigate these changes. Modified atmosphere packaging (MAP) also protects cut flowers from wilting (Bishop et al., 2007; Biji et al., 2015; Ghaani et al., 2016; Salgado et al., 2021; Kumar et al., 2022).

In addition, MAP, a well-validated horticultural commodity shelf-life extender and postharvest quality preservation technique, extends the shelf-life of fresh produce postharvest.

Also, a MAP design optimizes oxygen uptake and CO_2_ evolution in packaged fresh produce, as well as gas transfer through packaging films, by decreasing oxygen levels and increasing carbon dioxide levels in the package atmosphere. Microorganisms that cause spoilage can be controlled in MAP environments by reducing mold growth and flower decay. Furthermore, MAP reduces exposure to mold spores and other environmental contaminants (Belay et al., 2016). CO_2_, O_2_, N_2_, water vapor, and other trace gases are changed and selectively controlled to increase shelf life. Using this definition, there are no controlled atmosphere packaging (CAP) systems in commercial use. However, the combination of in-package or in-film O_2_ and C_2_H_4_ absorbers, together with CO_2_ release agents, could be classed as CAP, at least during the early stages of the storage life of the packaged product (Belay et al., 2016). Additionally, intelligent packaging technologies enhance product safety, durability, and quality standards in the food industry. With this system, product quality can be continually monitored and its status relayed to customers. As a result, customer satisfaction can be increased and product waste can be decreased. Intelligent packaging technologies are classified into three major categories: indicators, data carriers, and sensors. Despite its potential, intelligent packaging remains underutilized. Moreover, the delicate nature of cut flowers also makes them difficult to store (Popa et al., 2019). Therefore, fresh-cut flowers require knowledge of modern packaging technology to preserve them during storage and transportation. This review discusses the preservation aspects of fresh-cut flowers from a fresh-keeping perspective, which could prove beneficial for those in the floral industry. Additionally, it discusses the prospects for an integrated modeling approach and recent advances in modern packaging technology.

This understanding can inform the performance of various packaging formats. Consequently, there will be an increase in flower life and a higher quality of product. The current review suggests that future research should aim to identify the most effective packaging strategies for cut flowers.

## 2 Classical packaging

A package constitutes a significant influence over consumers’ perceptions and enhances their purchase. Inadequate harvesting, handling, and storage can cause significant losses in the plant’s production investment (Belay et al., 2016; Dias et al., 2017).

Furthermore, packaging materials must be resilient to vibration, shock, drop, compression, and refrigeration during transportation and storage to ensure the quality of flowers. To ensure compliance with the importing countries’ regulations, package standards vary depending on the species, cultivars, transportation mode, storage, and market outlet. In addition to being strong enough, the packaging material should be low cost or reusable, moisture-resistant, and easy to operate. Also, the most widely used packaging materials are cellophane, cardboard, butter paper, polyethylene, polypropylene, and polyolefin. Furthermore, to furnish relevant information about flower content, all types must be labeled (Wang and Qi, 1997; Patil, 2009; Gupta and Dubey, 2018; Kumar et al., 2022). In order to maintain the quality of flowers throughout the marketing process, packaging materials must maintain their physical, physiological, and pathological integrity. It is recommended to package orchid flowers in two-piece boxes and to keep ethylene scrubbers in the box as well. A preservative solution is poured into flasks containing single *Cymbidium* flowers packed in fern leaves. A three-sided box with a display window is used for packing the flasks (Jawaharlal et al., 2006). Also, the vase life of *Cymbidium* flowers packaged with cellophane paper is 28 days to 40 days, compared to an 18-day to 27-day vase life for unpackaged flowers. Furthermore, in comparison with the control (50 days without packing), flowers packed in cellophane had the longest vase life (56 days), followed by polypropylene (54 days) and low-density polythene (54 days) (De and Singh, 2016).

**Figure 1:**
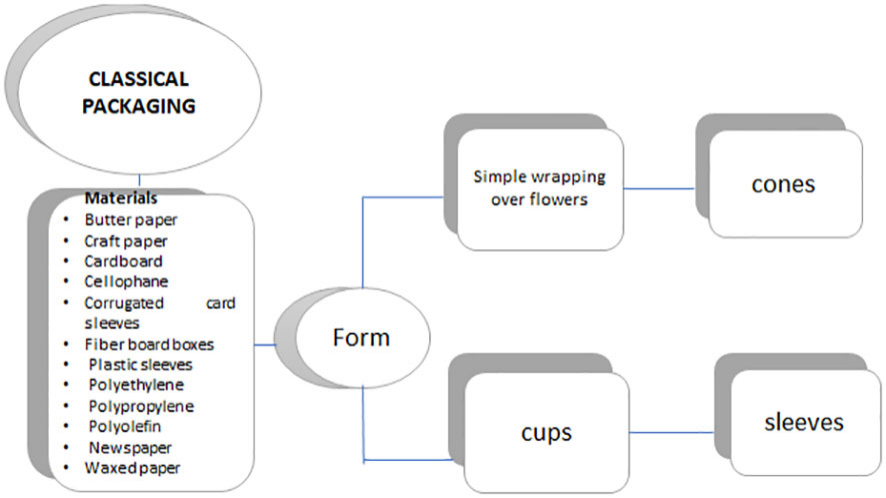
Classical Packaging materials for cut flowers.

In addition, marigolds are often packaged in newspaper, cellophane, and polyethylene. However, cardboard boxes lined with newspaper and cellophane showed the highest moisture content, maximum freshness index, and minimal loss of weight, as well as the least amount of spoilage of flowers. Chrysanthemums are packaged similarly. Corrugated fiberboard boxes in vertical hampers marked for upright stacking are the most suitable material for packaging *Gladiolus* spikes. This configuration thus minimizes the potential for geotropic curvature, which adversely affects flower quality due to gravity-induced damage. Corrugated card sleeves, waxed paper, or cardboard boxes are the most common packaging used for rose bouquets. Flowers are not chipped inside plastic sleeves to prevent humidity buildup. Packaging of potted flowering plants, such as potted *Hibiscus*, varies based on the size of the plant, the quantity of foliage, and the flexibility and fragility of the leaves and branches. Their packaging is also influenced by factors including their entanglement potential and injury risk during handling, transportation, and shipment. Historically, potted plants have been commonly packaged in clear plastic sleeves, craft paper, or fiberboard boxes wrapped in plastic or paper, with dividers between plants (**Figure 1**) (Twede et al., 2015).

In addition, traditional floral packaging failed to meet consumers’ demands for quality. Consequently, novel packaging strategies have become vital in expanding the production of various cut flowers. They have been employed to preserve flower quality, ensure microbial safety, and prolong vase life, presenting alternatives to traditional packaging methods. Furthermore, the aim of novel packaging is to improve certain aspects of packaging, such as efficiency, sustainability, quality, and protection, through the use of advanced materials and technologies. Moreover, numerous innovative packaging techniques exist, including MAP, CAP, intelligent packaging, and nanotechnology packaging.

### 2.1 Modified atmosphere packaging

MAP technology significantly contributes to maintaining the quality of cut flowers postharvest by delaying physiological changes (Poonsri, 2017). Furthermore, the elevated CO_2_ concentration within the packaging substantially diminishes respiration and ethylene synthesis, thereby significantly reducing the energy produced. The packaging permits the removal of undesirable gases and provides the means of manipulating oxygen levels and carbon dioxide concentrations. MAP effectively preserves the quality of fresh produce by reducing respiration intensity, material consumption, carbon dioxide production, oxygen consumption, and heat generation (Watkins, 2000; Sousa-Gallagher et al., 2013). In addition, MAP stabilization can be achieved by packing cut flowers in plastic of varying permeability to CO_2_ and O_2_. Nonetheless, the final concentrations of CO_2_ and O_2_ within the pack during storage, as well as the maintenance of water balance in the flowering stem, significantly influence the floral response.

Regulation of ethylene gas production will extend the storage duration for flowers, ensuring their quality and enhancing their lifespan. Although refrigeration and humidity control can significantly decelerate senescence and decay, they do not stop ethylene production. The term “modified atmosphere storage” (MAS) encapsulates storage under such atmospheric alteration. MAS offers several benefits, including ease of use, cost-effectiveness, and less complexity than controlled atmosphere and low-pressure storage methods. Despite these advantages, MAS lacks the precision of CAS (Ben-Yehoshua et al., 2005; Sandhya, 2010; Wilson et al., 2019).

**Figure 2:**
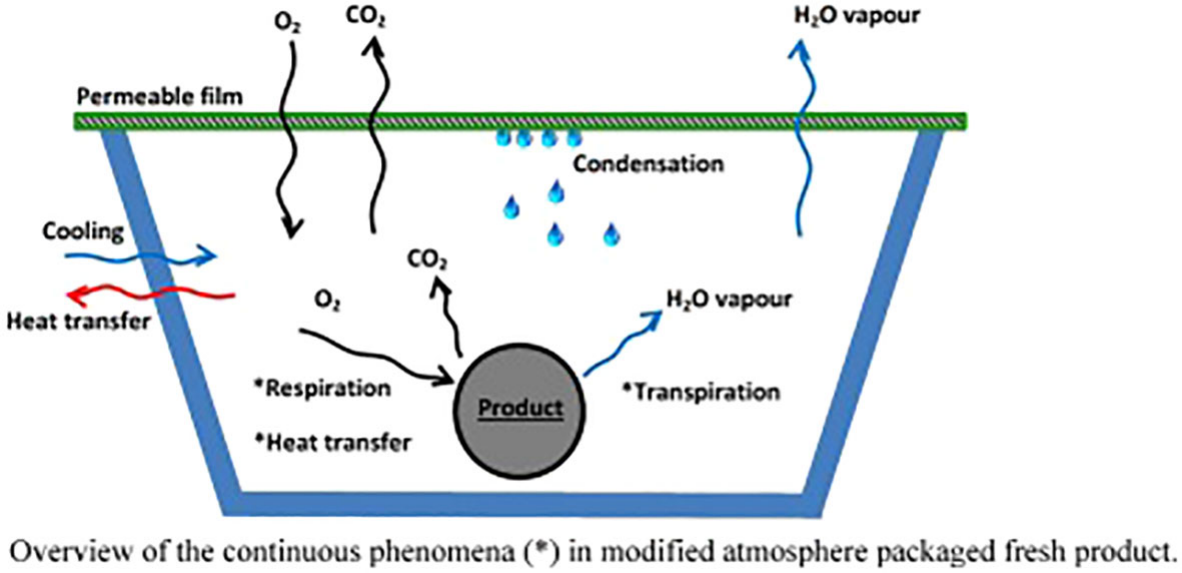
Overall heat transfer process in a modified atmosphere packaged fresh product. *Continuous phenomena within and around the packaged product (Belay et al., 2016).

On the other hand, the process of senescence in plants is a complex, highly regulated process. This involves a decline in photosynthesis, with the loss of chlorophyll and the dismantling of chloroplasts, as well as the degradation of macromolecules such as proteins, nucleic acids, and lipids. As a result, there is a significant difference between other flower systems in terms of nutrient mobilization and senescence mechanisms (“ethylene sensitive” and “ethylene insensitive”). These distinctions could be attributed in part to higher free SPD levels, which bind to intracellular constitutive molecules like DNA, thereby stabilizing their structures and slowing the progression of senescence. The PCA-soluble fraction is the primary source of conjugated PAs when irreversible senescence is caused by the ethylene burst (Buchanan-Wollaston, 1997; Bagni and Tassoni, 2006). It is reported that low levels of oxygen inhibit ethylene. The effect of CO_2_ on ethylene may also be reduced; it may act as a competitive inhibitor although it seems unlikely to bind to the ethylene receptor when examined closer (De Wild et al., 2003; Ben-Yehoshua et al., 2005). In 2000, Watkins described the importance of CO_2_ during MAP, demonstrating that CO_2_ affects several metabolic processes, such as respiration and 1-aminocyclopropane-1-carboxylic acid synthase (ACC). Physiological breakdown is a common consequence of exposure to low oxygen (1%) and high carbon dioxide (>20%) levels in numerous fresh vegetables. It is important to keep cut flowers away from chilling temperatures. Hence, to avoid chilling injury signs, CO_2_ accumulation and O_2_ reduction are helpful (Wang and Qi, 1997). It is generated by balancing the gases within the package in a dynamic way. As a result, fresh flowers must get enough oxygen to be consumed by the package. It is also necessary to have an equal output and production of CO_2_ (Smith et al., 2003; Sandhya, 2010). Depending on the specific permeability of these films, holes may be needed for a stable atmosphere inside (Del-Valle et al., 2003). It has been proven that MAP technology can prolong the storage period of fresh produce without affecting its natural quality postharvest. It is necessary to integrate the dynamic properties of the produce, packaging characteristics, and ideal equilibrium conditions for its environment to obtain an optimal MAP design (**Figure 2**) (Belay et al., 2016).

Escalona et al. (2007) have shown that MAP technology effectively extends the shelf life of fruits and vegetables. However, there have been few reports on cut flowers published. In addition, several postharvest changes negatively influence both the commercial value and vase life of cut flowers. These alterations encompass petal discoloration, petal detachment, tissue browning, and wilting. MAP manipulates the CO_2_ and O_2_ levels within the package to inhibit these changes (Poonsri, 2021). It is important to note that flower species differ considerably in their responses to altered circumstances during storage (Zeltzer et al., 2001). Furthermore, MAP techniques have demonstrated promising results when it comes to maintaining the quality and increasing the shelf life of some cut flowers, including carnations (Bishop et al., 2007), roses (De Pascale et al., 2005; Bishop et al., 2007), and lily (Wu et al., 2013). However, MAP has previously been reported to have a disappointing effect on cut flowers (Reid, 2001; Reid and Jiang, 2012). Furthermore, significant tissue browning, collapse, and decay were observed in *Trapaeolum majus* L., *Borago officinalis* L., and *Viola tricolor* L. stored at −2.5°C to 20°C in polyethylene bags (Kelley et al., 2003).

**Figure 3:**
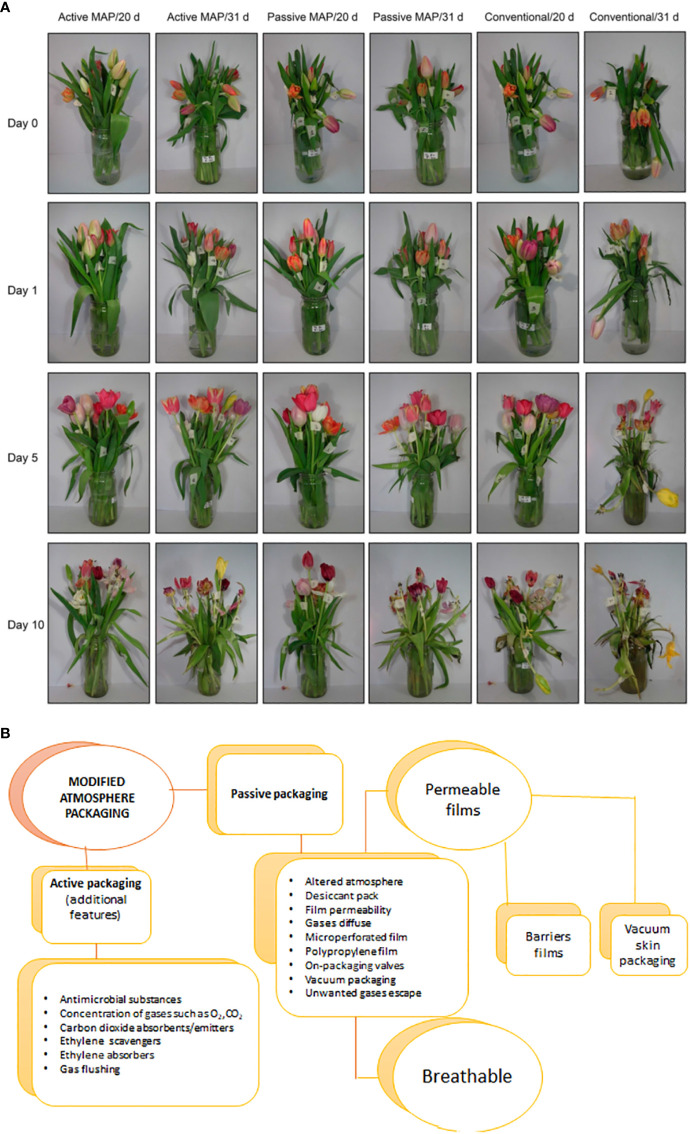
**(A)** Pictures of the evolution of the vase life of 10 tulip flowers each (composed of a mixture of eight cultivars) after storage at 0°C for 20 days and 31 days using active and passive modified atmosphere packaging (MAP) and cellulose film (conventional) (Aros et al., 2017). **(B)** Modified Atmosphere Packaging materials and their usage.

The storage of various flowers, including carnations, gypsophilas, solidagos, and several cultivars of roses, in MAP (7.09 kPa CO_2_ and 13.17 kPa O_2_) at 2°C for 10 days resulted in a quick increase in CO_2_ levels and a subsequent decrease in O_2_ levels. Consequently, the flowers preserved a significantly higher fresh weight (3.5 times) than flowers kept in a standard environment. MAP flowers have demonstrated a 10% reduction in water loss and a superior appearance after shipment in comparison to conventional carton flowers. As MAP shipments progressed, CO_2_ concentrations increased to 6%, while oxygen concentrations declined to 15%. During the same period, from day 1 to day 8, CO_2_ concentrations remained constant, while oxygen concentrations declined steadily. The application of the PAP system demonstrated its ability to delay senescence in flowers and leaves while also reducing ethylene biosynthesis. MAP storage at 5°C, using polypropylene (24) and high-density polyethylene (24), was found to enhance the preservation of *Gerbera* flowers. A vase life evaluation of flowers stored at PAP revealed reduced weight loss, high water uptake, and larger flowers than those not stored at PAP (Patel and Singh, 2009). Furthermore, the MAP system was found to extend the lifespan of tulip flowers significantly when stored at 0°C for 20 days. A floral arrangement stored in MAP outperformed one packaged in regular packaging, as demonstrated in **Figure 3** (Aros et al., 2017).

In addition, the lifespan of *Gladiolus* spikes was successfully extended when stored at 3°C–4°C, for 18 days, in either polypropylene or low-density polyethylene (Jhanji and Dhatt, 2017). The structure of the flower affects the efficiency of MAP. The application of MAP treatment to carnations, stored for 7 days at a temperature of 5°C, resulted in significant reductions in weight loss, decay, and visual quality deterioration (Kou et al., 2012). Conversely, the same treatment negatively affected the visual quality of snapdragons and increased weight loss (Kou et al., 2012, Kou et al., 2012). In addition, marigold flowers (*Tagetes erecta* L.) stored in MAP at 23°C for 8 days exhibited significant weight loss reduction and color preservation (Pal et al., 2016). The MAP treatment utilized polyethylene films, low-density polyethylene films, high-density polyethylene films, polyvinyl chloride films, and polypropylene films. All films were kept at 13°C and 95% relative humidity. MAP films significantly decrease anthocyanin content, respiration rates, and weight loss. Polypropylene film, among the conventional packing types, demonstrated the longest storage life, at 18 days, while ordinary conventional packaging had the shortest, at 9 days (Yimyong and Soni, 2014). It was possible to store spikes of *Ornithogalum* for up to 3 days at 4°C in MAP with cellophane (Yimyong and Soni, 2014). From the aforementioned findings, it is evident that the adaptability of various flower species to the MAP environment varies.

It can be divided into two types based on Montanez et al. (2010):

1. Active MAP: It consists of an evacuated package with the flowers inside and an infusion of the desired gas composition.
2. Passive MAP: Traditional methods are generally employed during the initial stages of packaging, which are subsequently substituted with more advanced methods. The permeability of a film and the rate of flower respiration are major contributors to this phenomenon. MAPs exert an influence on a process in both active and passive forms, primarily influencing gas transmission through the packaging (Moradinezhad and Dorostkar, 2021).

#### 2.1.1 Active MAP of cut flowers

In recent times, passive MAP has been utilized to considerably extend the vase lifespan of cut flowers. Fresh flowers cannot be stored and distributed in this way because of their limitations regarding maintaining the desired environment. Therefore, the shortcomings of passive packaging are being rectified via the active MAP method. This method (MAP) involves absorbing or scavenging undesirable compounds like CO_2_ and O_2_, removing excessive water, and scavenging ethylene and antibacterial substances. Other active systems may release or introduce into the package headspace compounds such as preservatives, CO_2_, antioxidants, and other chemicals (Yousuf et al., 2018).

The active MAP technique employs the flushing of packages with a gas mixture whose composition varies depending on the respiration rate and permeability of the film (Ghidelli and Pérez-Gago, 2018). In addition, a technology that embeds an ethylene scavenger into active packaging could prolong the shelf lives of fruits, vegetables, and cut flowers. An ethylene scavenger can be either an absorber (which absorbs and entraps ethylene) or a scavenger (which absorbs water through the chemical reaction between two materials) (Gaikwad et al., 2020). Furthermore, antimicrobial packaging enhances food’s nutritional and sensory qualities, with minimal preservation techniques needed (Vermeiren et al., 2000; Sung et al., 2013). In addition to failing to demonstrate benefits, commercial trials lack valid controls, which have clouded the results’ credibility. There have been many studies looking at the effectiveness of sealed packages (such as salad packages) for single flowers and small bouquets, but while the results have sometimes been promising, they are limited to a small number of flowers or even a few specific species and therefore have very limited general application (Reid and Jiang, 2012). Research has been conducted to elucidate the reasons behind unsuccessful implementations of controlled atmosphere storage (Yahia, 2009). Moreover, as temperatures rise (10°C, 15°C), petals show a decrease in respiration. Technological advancements can broaden the application of controlled atmosphere (CA) to fresh flowers, enhancing cost-effectiveness during transportation and storage (Kader, 2003).

In another study, a MAP film filled with 5% CO_2_ and 2% O_2_ was wrapped around orchid flowers in an active MAP experiment. In contrast, orchid flowers were stored passively without CO_2_ or oxygen under passive MAP conditions. The orchid flowers were stored at a temperature of 13°C and a relative humidity of 95% in the dark. In active MAP, orchid flowers were stored for 9.33 days. In normal atmosphere conditions, orchid flowers are stored for 7 days (0.03% CO_2_ and 21% O_2_) (Poonsri, 2021).

#### 2.1.2 Passive modified atmosphere packaging for cut flowers

The rate of respiration and film permeability are key factors influencing the impact of passive MAP (Ghidelli and Pérez-Gago, 2018). In the process of passive modified atmosphere (MAP) storage, gases are partially modified while in storage, rather than being completely altered. MAP is helpful aside from reducing respiration. Despite their widespread use in commercial applications, such as cut flower storage, they have not achieved widespread commercial success. Several studies suggest an increased lifespan postharvest for *Gladiolus* and cut roses although these findings have yet to be substantiated through commercial trials. Packaging materials play a passive role in preserving a product’s freshness. They naturally diffuse gases to create the desired atmosphere, and this can be achieved through the use of barrier packaging films. Diverse types of film materials are used to produce passive preservation. In a passive MAP state, significant quantities of carbon dioxide and reduced amounts of oxygen are produced over time due to a high respiration rate and gas permeability of the packaging film. Achieving optimal atmospheric conditions necessitates a balance in the permeability of the packaging film and the respiration rate of the product (Brody et al., 2010). Passive MAP retains flowers in plastic wrap without contact with CO_2_ or oxygen. Flowers are wrapped in MAP plastic wrap (without CO_2_ or oxygen) to preserve them. Poonsri (2021) reports that passive MAP flowers are wrapped in MAP plastic without carbon dioxide and oxygen (CO_2_ and O_2_). The experiments were executed at a temperature of 13°C, under 95% relative humidity. Furthermore, the biochemical parameters studied included anthocyanin content, protein degradation, amino acid metabolism, and electrolyte leakage, and the biochemical parameters studied included anthocyanin content, protein degradation, amino acid metabolism, and electrolyte leakage. In their study, Fadda et al. (2020) explored the effectiveness of passive MAP at 5°C in the chemical properties of *Calendula* flowers. The microperforated film used in MAP with microscopically shaped holes prevents bacterial growth and preserves the visual and nutritional quality of *Calendula* petals in storage at 5°C for up to 10 days. It can be concluded that low humidity, despite reducing weight, adversely affects flower freshness and hastens the degradation of total phenols and carotenoids. In a study, Patel and Singh (2009) conducted an experiment to determine the effects of passive MAP and storage temperature on the quality of *Gerbera* flowers. The application of polypropylene and high-density polyethylene passive MAPs at 5°C significantly reduced vase bending and weight loss. These outcomes paved the way for extended vase life and postponed petal senescence, attributable to polypropylene and high-density polyethylene MAPs.

According to the study of Yahia (2009), orchids stored in PP packaging exhibited the longest average storage life at 15.66 days. Decreasing oxygen levels during storage could induce elevated levels of carbon dioxide, thereby affecting ethanol production and respiration rate. Upon the successful completion of the control experiment after 6 days, ethylene levels steadied at 13.22 ppm, leading to wilting, abscission, and senescence (Poonsri, 2020). Cut flowers are said to have an extended shelf life when passive MAP is used in conjunction with low temperatures, as reported by Yahia (2009) and Poonsri (2020).

**Figure 4:**
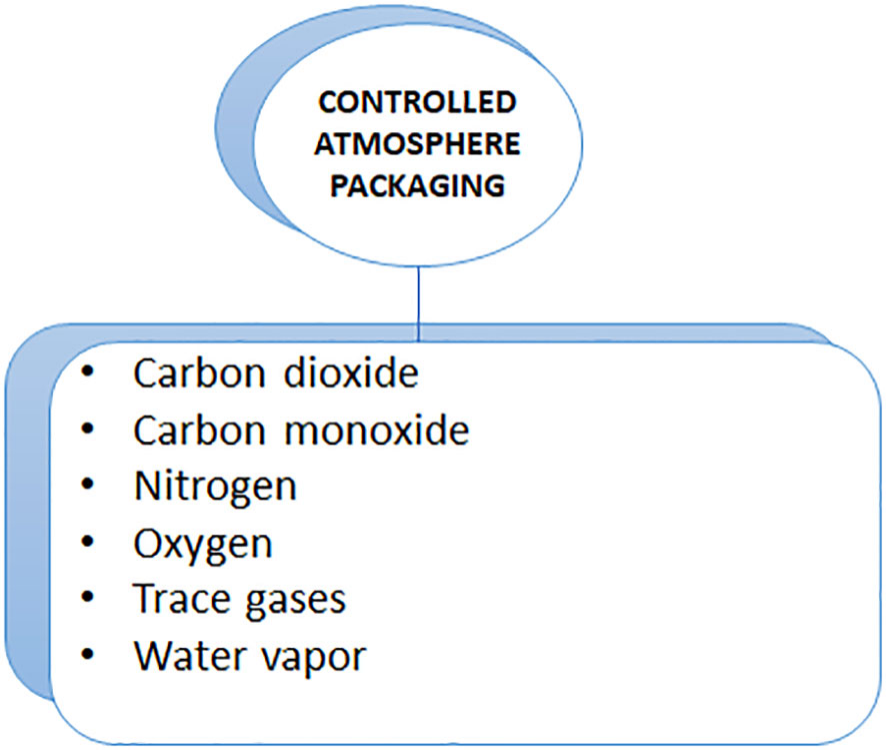
Controlled Atmosphere Packaging and their materials.

Additionally, Singh et al. (2009) analyzed the preservation of jasmine flower buds using polypropylene film packed under passive modified atmospheres (MAPs). Based on a comparison between MAP-stored and non-MAP-stored buds, buds stored in MAP appeared to retain greater freshness with a lower physiological loss of weight (PLW%) and a longer shelf life. The percentage of brown and wilted buds increased with storage temperatures as high as 10°C and storage times as long as 20 days. The temperature during storage influenced the freshness of a bud as well. Temperature is also a factor affecting the freshness of a bud. In both MAP-treated and MAP-less buds, freshness was completely lost after 10°C storage. Chrysargyris et al. (2018) reported that harvested *Tagetes* flowers survived for up to 14 days under passive modified atmosphere packaging (with or without ethanol) under different salinities (0, 50, and 100 mM). A 14-day storage period at 100 mM of NaCl reduced the weight of the flowers and changed their marketability. The presence of salt during harvest and/or ethanol during storage induces non-enzymatic mechanisms (e.g., proline content) as well as enzymatic mechanisms (catalase). In an experiment, scientists found that short-term exposure to salinity or ethanol may enhance carotenoid and anthocyanin levels, which makes them potentially beneficial nutraceuticals (**Figure 4**).

## 3 Controlled atmosphere packaging for cut flowers

Physiological changes and respiration can be slowed down in three ways. The first method is to lower the ambient temperature, the second method is to reduce the oxygen, and the third method is to increase the amount of carbon dioxide. In the MAP method, these three steps are also followed. However, there is one major difference between the MAP and the CAP: the *management* of changes throughout the flowers’ life (durability). In other words, the MAP method cannot control what happens to the product after packaging or respiration.

However, the best combination of retaining gases in the package is achieved by changing three gases. CA storage combined with refrigeration reduces respiration and delays yellowing and quality changes.

There are some functions for CA generation and maintenance, including O_2_ removal, excess CO_2_ removal, and the addition of air to replace O_2_ consumed by respiration. In addition, there is the removal of C_2_H_4_ and, in some cases, the addition of CO_2_. There are several factors to consider when selecting appropriate functions and devices for the generation and maintenance of CA, including the storage conditions required for the products and the location of storage. Scientists have shown that CA storage affects flower quality, including positive and negative, and may also have no effects. This includes reductions in physiological processes consisting of C_2_H_4_ production, respiration rate, volatile compounds, phytochemical compounds, color, etc. However, tolerance for individual flower varieties needs to be considered (Patel and Singh, 2009; Bodbodak and Moshfeghifar, 2016; Singh et al., 2021).

The benefits of controlled atmosphere packaging (CAP) are as follows:

a) Carbon dioxide (CO_2_) levels are increased preventing mold and bacteria growth.
b) The amount of oxygen (O_2_) in the atmosphere decreases.
c) Monitoring nitrogen levels: Nitrogen is an inert gas that inhibits respiration and enzyme activity.
d) It creates a low storage temperature.
e) It prevents high levels of internal ethylene from creating senescence and death-inducing changes.

Controlled atmosphere (CA) storage significantly impacts the postharvest quality and marketability of cut flowers. Increased CO_2_ concentration leads to a reduction in respiration rate, which benefits product quality maintenance (Burana et al., 2015). One of the primary limitations of CA storage of flowers is the fluctuation in optimal levels of CO_2_ and O_2_ required for various flowers (Gupta and Dubey, 2018). Moreover, an environment of low O_2_ concentration can also decelerate the respiration rate, thereby preserving flower quality (Cefola et al., 2016). In addition, under controlled atmosphere storage, changes in metabolism patterns, respiratory enzymes, and membranes were substantially delayed, inhibiting the premature senescence of cut flowers (Defilippi et al., 2006). It appears that there have been a limited number of studies examining controlled atmosphere storage of cut flowers. Further research is needed to explore the relationship between controlled atmosphere packaging and flower cooling. Globally, research in this sector is limited, necessitating the development of advanced postharvest technology, including cold storage and controlled atmosphere packaging for cut flowers. A controlled atmosphere serves as a complement to refrigeration, thus enhancing the efficiency of refrigerated storage (Akbudak et al., 2005).

Dias et al. (2017) demonstrated that CA containing 3% O_2_ and 6% CO_2_ at 1°C maintained a significant quality for storage periods between 14 and 21 days. It is suitable for cut rose storage under a controlled atmosphere and could also be used for rose export procedures. CA concentrations were associated with higher flower quality, longer green foliage, and a lesser incidence of *Botrytis cinerea*. As a result, this indicates that the conditions for storing cut roses are adequate.

As part of their study, Burana et al. (2015) assessed how short-term CA affected the vase life of cut carnations, prairie gentian, and chrysanthemum flowers at 5°C with elevated CO_2_ concentrations of 10%, 15%, or 20%, followed by 23°C water following senescence. Based on this study, short-term CA can decrease ethylene production and prolong vase life. It would be helpful to conduct more studies in order to optimize the levels, durations, and temperatures of CO_2_ treatment. A study by De and Singh (2016) found that in CA storage, orchid flowers are stored in gas-tight cooling chambers using cooling systems designed to produce *a higher level of CO2 and a lower level of oxygen in order to reduce respiration rates, ethylene production, and reactions.* It is usually recommended to keep CO_2_ storage levels above 4% and not below 0.4%.

Furthermore, in CA storage, the gaseous mixture composition is altered and stored differently from that of the ambient atmosphere. It is generally recommended to maintain cut flower quality in a storage unit by retaining CO_2_ above 1% and oxygen below 8%. In addition, it is recommended to maintain a specified temperature and relative humidity. Ethylene production and respiration rates are reduced when the concentration of these gases is modified within the tolerance level of each species. Concentrations that are too high or too low, however, may increase decay and senescence. There has been little use of CA storage for storing cut flowers. CA storage for cut flowers has only been reported in a few studies, including the study by Saltveit (1997). It has been shown that in the presence of elevated CO_2_/low oxygen atmospheres, fresh flowers are delayed from undergoing undesirable postharvest changes, such as wilting and aging. In a controlled atmosphere at 2°C, cut Red Gala rose flowers lasted 45 days in a controlled atmosphere (5% CO_2_ and 4% O_2_). A controlled atmosphere kept at 2°C (5% CO_2_ and 4% O_2_) for 45 days retained red Gala roses’ vase life slightly longer than air-stored roses, as reported by Poonsri (2015). Recent advances in CA storage technology have led to several improvements. In some polymeric packaging films and containers, certain additives are used to modify the headspace atmosphere. The CA storage technology has undergone several improvements in recent years. Headspace atmospheres can sometimes be modified with the addition of certain additives to polymeric packaging film or containers. Active modified atmosphere packaging describes this type of packaging. It was developed so passive MAP deficiencies can be eliminated through active MAP. It is possible to use an oxygen scavenger when a film is effective as a moisture barrier but not as an oxygen barrier. Pack oxygen will be excluded in this way. In the same way, MAP can be controlled by carbon dioxide absorbents and emitters as well as ethanol emitters and ethylene absorbents. Flowers are placed along with appropriate absorbent materials. In addition to modifying package headspace, they also extend the flowers’ shelf life. It is believed that low internal oxygen concentrations have beneficial effects on reduced respiration and reduced ethylene sensitivity (largely due to elevated CO_2_ levels). CA can be established rapidly (0.7% to 1.5%), ethylene-free, and programmed (or sequential) (such as storing in 1% oxygen for 2 to 6 weeks followed by 2% to 3% oxygen for the rest of the storage period) when low O_2_ concentrations (0.7% to 1.5%) are accurately monitored and controlled. Kader (2003) explored dynamic CA in which the levels of CO_2_ and O_2_ are adjusted based on monitoring some attributes.

A summary of most studies that focused on the responses of plant species of cut flowers to different packaging and average storage life are listed in **Table 1**.

**Table 1:** Responses of plant species of cut flowers to different packaging and average storage life.

No.	Crop	Type of package	Storage hours/days	Temperature	Responses to package	Source
**1. **	Calendula	Passive modified atmosphere packaging (MAP) with microperforated film	10 days	5°C	Overall (visual and nutraceutical) quality is good	Fadda et al. (2020)
**2. **	Carnation and snapdragons	Modified atmosphere packaging (MAP) sealed with a gas-permeable film	7 days	5°C	Reduced weight loss and decay incidence and maintained visual quality	Kou et al. (2012)
**3. **	Carnation, prairie gentian, chrysanthemum	Short-term controlled atmosphere	2 h	5°C and 23°C	Prolong the vase life of ethylene-sensitive flowers	Burana et al. (2015)
**4. **	Cultivars of roses	Modified atmosphere plastic containers (7.09 kPa CO_2_ and 13.17 kPa O_2_)	10 days	2°C	Flower quality was good	Zeltzer et al. (2001)
**5. **	*Dendrobium* orchids	Modified atmosphere packaging (5% CO_2_ 2% O_2_), Controlled atmosphere Normal package (NP)	28.33 days 18.15 days 11.67 days		Delaying the senescence	Poonsri (2015) Poonsri (2017) Poonsri (2020)
**6. **	*Gerbera*	Passive MAP	7 days	5°C, 10°C, and 15°C	Significantly lower physiological loss in weight; improved flower size, petal length, and width during vase life	Patel and Singh (2009)
**7. **	Jasmine	Polypropylene packaging film for passive MAP (MAP)	10 days	2°C	Physiological reduction in weight loss	Singh et al. (2009)
**8. **	Marigold Tagetes	Passive modified atmosphere packaging (with or without ethanol), when exposed to salinity (0, 50, and 100 mM NaCl)	14 days		Flowers survived, increasing the levels of carotenoids and anthocyanins, making them potential nutraceuticals	Chrysargyris et al. (2018)
**9. **	Marigold	Low-density polyethylene (LDPE) bags,	8 days of storage	23°C	Significantly reduced weight loss and retained color and overall appearance	Pal et al. (2016)
**10. **	Orchid	Passive MAP, polypropylene packaging, increase in CO_2_ and a decrease in O_2_ inside the packaging	15.66 days		Longest average storage life	Yahia and Singh (2009)
**11. **	Orchid	Active MAP, the orchid flowers were wrapped with MAP film filled with 5% CO_2_ and 2% O_2_	9.33 days		Flower size is longer	Poonsri (2021)
**12. **	*Ornithogalum* spikes	Modified atmosphere packaging with cellophane	3 days	4°C	Best for storage	Dastagiri et al. (2014)
**13. **	Red Gala rose	The flowers were stored in a controlled atmosphere (5% CO_2_ and 4% O_2_)	45 days	2°C	Longer vase life	Poonsri (2015)
**14. **	Roses ‘Avalanche’	Controlled atmosphere (3% O_2_ and 6% CO_2_)	14 days and 21 days	1°C	Significantly higher flower quality, longer green foliage, and minor *Botrytis cinerea* incidence	Dias et al. (2017)
**15. **	Tulip	Modified atmosphere packaging	20 days	0°C	Significantly better and successfully extended postharvest life	Aros et al. (2017)

## 4 Future generation of packaging materials for cut flowers

### 4.1 Nanotechnology packaging

In modern times, passive or traditional flower packaging is being replaced by innovative, interactive, and responsive flower packaging designed with nanotechnology. Several factors have contributed to the development of smart packages aimed at extending flower shelf life, such as environmental awareness, advances in new knowledge (such as nanotechnology and biotechnology), and consumer lifestyle changes in flower production, sales practices, and consumer lifestyles. Mlalila et al. (2016) and Salgado et al. (2021) observed that it is important to maintain innocuousness and quality while also caring for the environment.

The utilization of nanotechnology in the synthesis of nanoparticles has paved the way for their use in food packaging. Consequently, it is expected to enhance the properties of food and flower packaging, enabling them to retain freshness for a longer time. Significant changes in packaging material properties have been achieved using nanoparticles. Commercially, a variety of nano-based packaging materials are also available, along with enhanced, active, and intelligent packaging, guaranteeing food quality and traceability. The ability of nanoparticles to scavenge oxygen, be UV-impervious, and possess antimicrobial properties makes them excellent candidates for use in nanocomposites. Due to their notable surface area to volume ratio, nanoparticles can be toxic. Hence, it is imperative to understand their migration and interaction with polymer matrices when developing packaging materials (Ashfaq et al., 2022).

Nanomanufacturing is the process of manufacturing, manipulating, identifying, and producing nanomaterials (1–100 nm). Packaging polymers can be modified to improve their strength, durability, flexibility, barrier, reuse properties, and durability by adding additives. This technique can significantly extend the shelf life of food products and cut flowers. Nanotechnology has recently found its way into the food industry, where it is being utilized to develop innovative packaging materials. Nanoparticles (NPs) can be used as reinforcements to improve the barrier and mechanical properties of polymers. The application of nanoparticles to biopolymers reduces the demand for raw materials (more sustainable), thereby reducing dependence on petroleum (Bumbudsanpharoke et al., 2015; Souza and Fernando, 2016; Cerqueira et al., 2018a; 2018b; Shukla et al., 2019).

#### 4.1.1 The most commonly used nanoparticles in food packaging and its potential to be used as flower packaging

Integration of nanotechnology with traditional food packaging materials, such as films and containers, can preserve food quality and extend shelf life (Tsagkaris et al., 2018). In addition, nanotechnology can be employed to create superior packaging, characterized by improved physical, mechanical, and antibacterial properties. Moreover, nanotechnology facilitates the development of smart packaging, which can be monitored and controlled to maintain ideal conditions for food products (Primožič et al., 2021). Commercially available packaging materials are now used to package cut flowers, but additional research is needed to enhance their competitiveness.

The use of nanotechnology in food contact materials (FCMs) is the largest current application of this technology in the food sector and is divided into four main categories by Chaudhry et al. (2008): i) FCMs with improved packaging properties (gas barrier, mechanical, etc.) because of the incorporation of NPs; ii) “active” FCMs that gain additional properties such as antimicrobial or oxygen scavenging through the incorporation of NPs; iii) “intelligent” FCMs as result of the incorporation of nanosensors; and iv) biodegradable polymer–nanomaterial composite biopolymers with improved characteristics using nanofillers.

**Figure 5:**
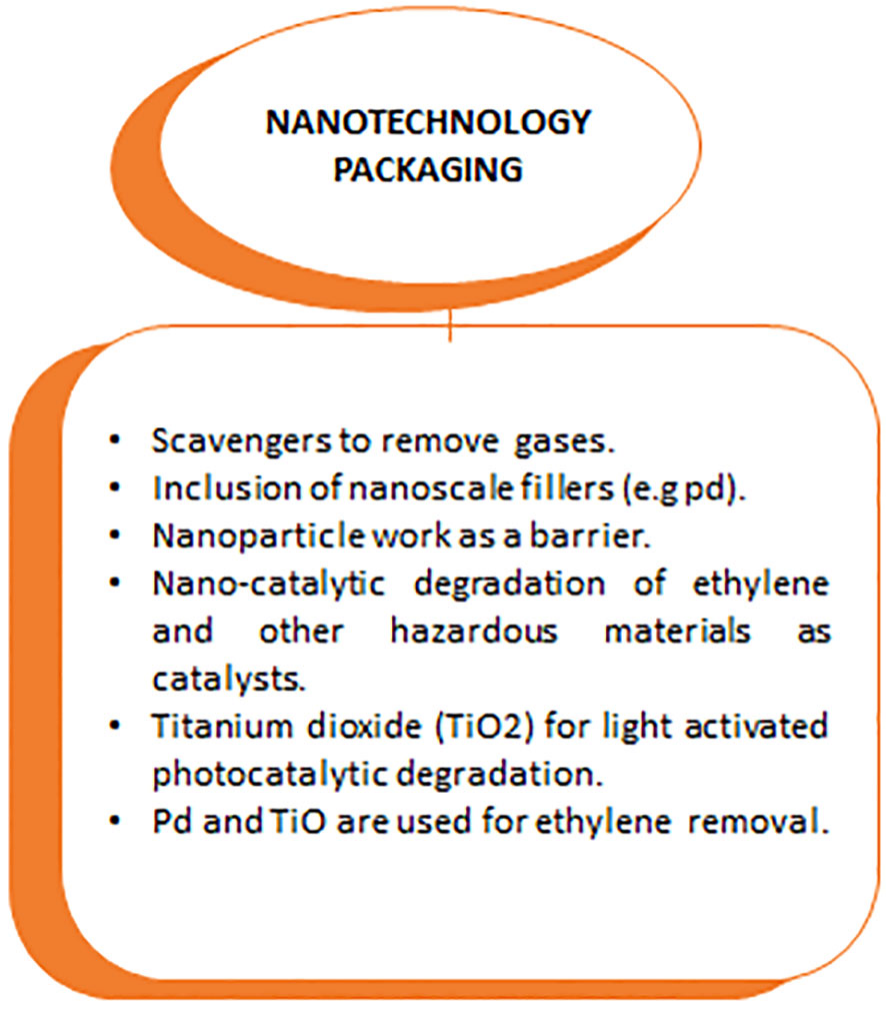
Nanotechnology Packaging’s materials and their usage.

Among the latest innovations in the food packaging industry, the use of biodegradable polymers reinforced with nanofillers is highlighted due to its sustainable appeal. This matches consumers’ demand for more environmentally friendly products. This is because they are sustainable and match consumers’ desire for environmentally friendly products (**Figure 5**) (Abdollahi et al., 2012; Tang et al., 2012).

### 4.2 Intelligent packaging

Gregor-Svetec (2018) predicts that smart packaging will continue to be the fastest-growing market in the future due to its unique, interactive, and customer-friendly features. Furthermore, packaging technologies must be innovative to address a variety of challenges. These technologies can also be applied for intelligent and active packaging of cut flowers. Flowers are usually packaged to meet four basic functions: protection or preservation, containment, convenience, and communication. As packaging grows smarter and more active, these basic functions are improved. Nanotechnology in food packaging produces clever, interactive, and responsive food packaging with enhanced functionalities that move from passive or traditional food packaging to active or innovative food packaging (Wilson, 2007; Mlalila et al., 2016; Gregor-Svetec, 2018).

In 2004, the European Commission defined intelligent packaging materials as “materials and articles that monitor the condition of packaged foods or the surrounding environment” (Ghaani et al., 2016). A thorough evolution is taking place in both of these fields, which leads to ever-changing definitions of intelligent and active packaging. Active packaging refers to a package that adjusts to suboptimal physiological or environmental conditions to improve them. In addition to barcodes, radio frequency identification tags (RFIDs), sensors, and indicators, intelligent packaging is also called smart packaging since it is equipped with smart devices for communicating, monitoring, sensing, recording, tracking, and indicating food safety, quality, and history during the supply chain (Wilson, 2007; Kalpana et al., 2019). To become more efficient and effective, intelligent packaging systems are being developed. A novel packaging idea will ensure the quality of packed food during shipping and storage by maintaining the food state (Kaushani et al., 2022).

In addition, intelligent packaging relies on the ability to communicate information gathered by the sensor. A variety of sensors have been developed based on chemical, enzymatic, immunochemical, or mechanical reactions. It is possible to place these sensors on or inside the package. Furthermore, communication of time and temperature conditions and history, oxygen and carbon dioxide levels, package leaks, spoilage, ripeness, freshness, microbial growth, and specific foodborne human pathogen identification can be accomplished and detected with these tools. In addition, nanosensors can be used to monitor the external and internal conditions of fresh and processed foods, an important application of nanotechnology (Wilson, 2007; Fuertes et al., 2015; Caon et al., 2017). One type of intelligent packaging is the use of freshness indicators, which are small devices printed on packaging materials or attached to package labels. It is usually possible to detect spoilage or the freshness of packaged goods by observing the color change of the packaging. Various quality-related metabolites, which are closely related to the type of product, microbial growth, packaging material, and storage conditions, were investigated by Butler (2012) and Fang et al. (2017).

Furthermore, an intelligent package responds to changing external or internal stimuli in response to an “on/off” switch, to communicate the product’s status to the consumer (Yam et al., 2005). The 2016 Strategic Research and Innovation Agenda recommends that the European Technology Platform (Food for Life) conducts research on smart intelligent/communicative packaging. A chromogenic marker’s simplicity has some advantages and some limitations, including a lack of specificity (false positives or false negatives). Also, poor quality is not necessarily associated with certain target metabolites. It is crucial to correlate metabolite concentrations with organoleptic quality and safety. Time–temperature indicators (TTIs) are also innovative chromogenic indicators. Intelligent packaging includes food packaging with temperature indicators. Real-time monitoring determines the impact of temperature on food quality (Kerry and Butler, 2008; Zhang et al., 2016).

**Figure 6:**
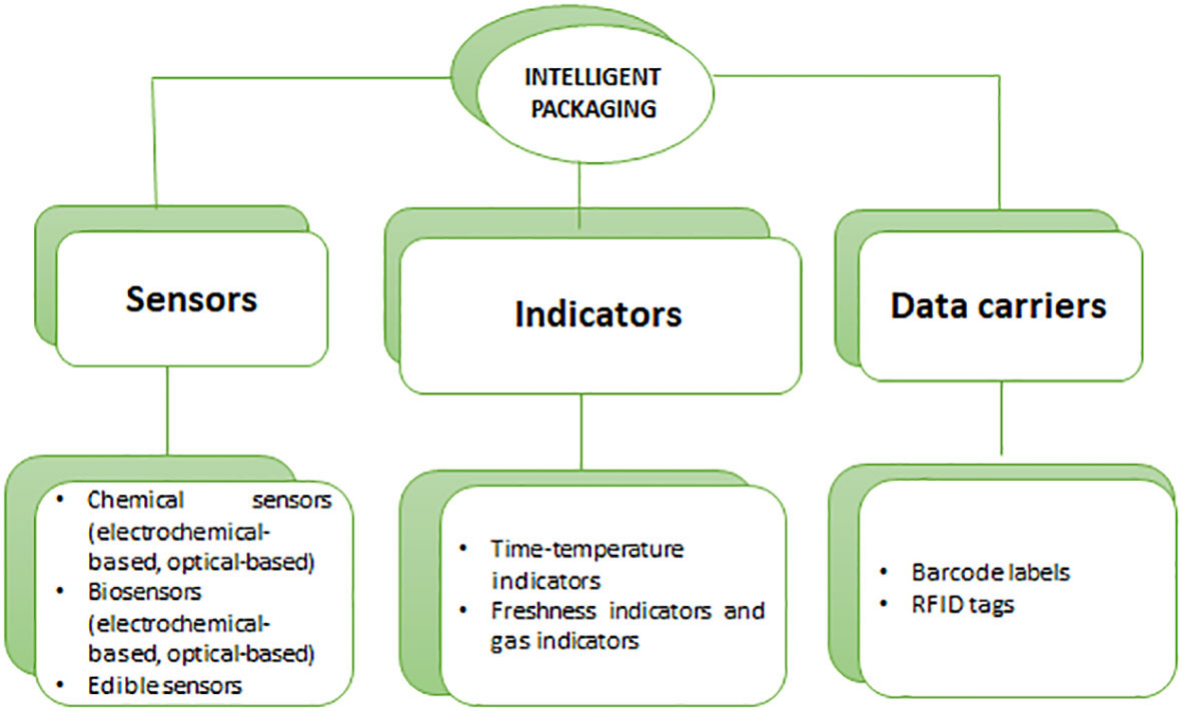
Intelligent Packaging materials and their usage.

Moreover, these three types of technologies are used in intelligent packaging, depending on the type of product. Intelligent packaging uses sensors, indicators (such as gas sensors, freshness indicators, and temperature indicators) (**Figure 6**), and radio frequency identification (Vanderroost et al., 2014; Müller and Schmid, 2019). Nopwinyuwong et al. revealed a packaging system (or part of the package) that continuously monitors metabolites related to food spoilage to determine food freshness. As a result of the decrease in pH, this indicator’s color changed from basic to acidic (Nopwinyuwong et al., 2010).

In addition, RFIDs and application-specific integrated circuit (ASICs) are used to identify and monitor temperatures. Intelligent packaging is now possible thanks to communication systems. Information about packages can be communicated through this technology. This technology allows packages to interact with people or appliances remotely to receive information about the condition of the packages. At present, wireless communication for packaging is costly, which limits its use in general. Despite this, costs remain low, and technology applications in the future should only be limited by our imaginations. An alternative to RFID packaging is ASIC technology. Having a custom-designed silicon chip, ASIC is considered a simpler and more economical technology. RFID technology has an economic threshold, which ASIC devices may overcome. Several studies are underway to deal with microbial contamination of flower containers using these two approaches. Scientists worldwide are investigating the release of volatile antimicrobial compounds into flower storage containers to minimize microbial growth. It is also possible to develop flower containers with antimicrobial compounds. To reduce microbial growth in flower storage containers, researchers are studying volatile antimicrobial compounds. Additionally, antimicrobial compounds are added to plastic plant containers. There has been a general problem with such technologies: flowers can become tainted if high enough concentrations of volatile antimicrobial compounds are administered in the headspace—even though they may also be effective in reducing microbial growth and extending flower freshness (Wilson, 2007).

Finally, intelligent packaging constitutes a burgeoning technology in the realm of flower packaging. Despite its nascent status and lack of full-scale commercial viability, intelligent packaging holds tremendous potential to enhance cut flower safety, quality, and traceability. Furthermore, its utility for consumers can be markedly enhanced. The system enables communication with the rose product and serves as a conduit for early warnings to the consumer, regardless of its lack of sensor capability for external or internal environment detection.

### 4.3 Green/sustainable packaging system in ornamental cut flowers

The concept of sustainable packaging integrates all phases of the supply chain and encompasses a wide range of eco-friendly materials, practices, and designs. To improve the quality of our ecosystem, we must reduce pollution due to plastics and other non-biodegradable items. Packaging sustainability is about more than just the product itself—it is about the entire supply chain. In addition to obtaining non-toxic materials and employing eco-friendly production methods, we will expedite the processing procedure, lower the cost of materials and labor, and use eco-friendly packaging.

Plastics and non-biodegradable packaging materials are becoming increasingly unpopular among customers today. It is essential to package flowers properly since they are perishable goods that must be kept at a certain temperature and hydrated during transportation. Additionally, businesses are developing eco-friendly and non-toxic containers and packages, such as polymeric formulations, which can last longer and be cross-contaminated-free. In the first layer, a biodegradable film rehydrates the flowers by utilizing moisture that has been drained from them. The film layer prevents moisture from accumulating in the flower stems and preserves oxygen levels in the flowers. In order to prevent excessive water use while transporting flowers, sustainable flower packing is the goal. Another goal is to be able to transport twice as many flowers per truck (Matthews et al., 2021).

**Figure 7:**
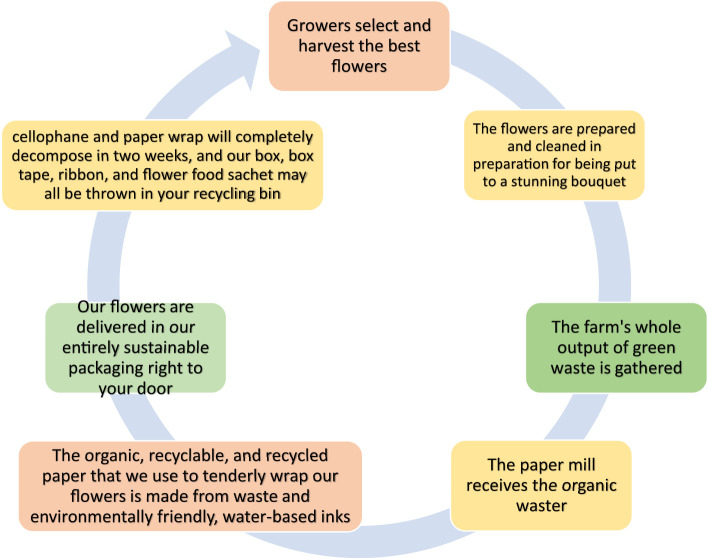
The closed-loop flower waste sustainable system.

The majority of paper packaging is made out of wood, a renewable resource. This substance is usually mixed with other materials, such as metal or plastic because it is easily polluted. As long as recyclable packaging is clean and does not cling to other materials, such as drink cartons, it can be transformed into fresh, reusable paper or cartons. The concept of circular packaging is related to sustainable packaging. The environment is protected without sacrificing quality with eco-friendly packaging. However, this still leads to linear processes, like burning to recycle. As shown in **Figure 7**, circular packaging keeps items and resources useful and valuable at all times. Therefore, when choosing a sustainable packaging material, it is important to consider the whole value chain (Amin et al., 2022; Ribeiro et al., 2024).

The adoption of biodegradable advanced packaging systems is crucial to keep up with the recent cut flower boom, including MAP, CAP, composite packaging, antimicrobial/antifungal packaging (AP), edible packaging (EP), and nano packaging (NP). Plastic-based films are minimized in favor of biodegradable and edible films and coatings as a result of stringent environmental legislation. A good example of an active packaging technology is MAP.

Primary packaging is made of plastic since it is light, flexible, and reasonably priced. Plastics, however, are not all recyclable. There are a variety of plastic polymers that make up most plastics. Recyclable plastics also consist of many layers, which complicates the recycling process. Multilayered plastic packaging includes oxygen barriers as well. In addition to providing flowers with the best protection possible, these packaging options minimize flower waste and prolong the vase life of the flowers. Moreover, raw materials are used more efficiently and CO_2_ emissions are reduced (Amin et al., 2022; Yadav et al., 2022; Malakar et al., 2023).

These techniques are rarely used for flower species. In addition to other physiological bottlenecks, this may be due to a lack of knowledge, negligence, and suppression. A novel or advanced packaging system that is resistant to chilling stress should be considered for international exports of cut flowers since it is one of the major setbacks.

## 4 Future perspectives

The optimization of each flower type is an essential prerequisite for an intelligent packaging system. For example, ASICs are being explored as a potential alternative to RFID packaging. This optimization would enhance the microbicidal effects of this technology, thereby improving treatment uniformity. Also, intelligent packaging provides information regarding quality and environmental changes within food, and cut flower information is centered on parameters such as time and temperature, which includes sensors or indicators for freshness, time–temperature, integrity, etc. Furthermore, the integration of appropriate cooling systems into the storage chamber is essential to minimize temperature buildup. More research is required in this area. Currently, there is no market for commercial packaging that promises pathogen removal from flowers. Significant progress has been made in these areas, but the benchmark for these technologies’ performance should remain high. The effectiveness of technologies that detect or eliminate pathogens should be guaranteed at all times for consumers to confidently rely on them to assess the safety of flowers they smell. Researchers working on intelligent and active packaging face challenges. The adoption of cut flower trading would not only enhance profitability but also stimulate scientific advancement.

## 5 Conclusions

As a concluding point, traditional packaging methods failed to meet the stringent consumer expectations for product quality. Instead, they focus on quality (maintaining flower freshness), safety (ensuring microbial control), and durability (extending vase life). A range of novel packaging techniques are available, such as MAP, CAP, intelligent packaging, and nanotechnology packaging. Innovative flower packaging today utilizes nanotechnology. With this technology, packaging becomes interactive and responsive, replacing traditional passive methods. Furthermore, severe environmental legislation has encouraged a shift away from plastic-based films in favor of biodegradable or edible alternatives. The significant environmental impact can be significantly mitigated through packaging that is either reused or recyclable.

## Author contributions

ME-M: Data curation, Formal analysis, Funding acquisition, Visualization, Writing – review & editing. NR: Conceptualization, Data curation, Project administration, Software, Supervision, Writing – original draft. SM: Data curation, Investigation, Methodology, Resources, Validation, Visualization, Writing – original draft. SA: Data curation, Investigation, Validation, Visualization, Writing – review & editing. TS: Conceptualization, Funding acquisition, Project administration, Software, Supervision, Writing – original draft.
